# Describing and Exploring Coping Strategies among Those Diagnosed with Cancer as an Adolescent or Young Adult: A YACPRIME Study

**DOI:** 10.3390/curroncol31020050

**Published:** 2024-01-24

**Authors:** Amanda Wurz, Anika Petrella, Joshua Tulk, Catherine M. Sabiston, Fiona Schulte, Jackie Bender, Norma D’Agostino, Sharon H. J. Hou, Geoff Eaton, Karine Chalifour, Sheila N. Garland

**Affiliations:** 1 School of Kinesiology, University of the Fraser Valley, Chilliwack, BC V2S 7M7, Canada; amanda.wurz@ufv.ca; 2 Cancer Clinical Trials Unit, University College Hospital, London NW1 2BU, UK; anika.r.petrella@gmail.com; 3 Department of Psychology, Faculty of Science, Memorial University, St. John’s, NL A1C 5S7, Canada; 4 Department of Exercise Sciences, Faculty of Kinesiology & Physical Education, University of Toronto, Toronto, ON M5R 0A3, Canada; catherine.sabiston@utoronto.ca; 5 Department of Oncology, Division of Psychosocial Oncology, Cumming School of Medicine, University of Calgary, Calgary, AB T2N 1N4, Canada; fsmschul@ucalgary.ca (F.S.); sharon.hou@ucalgary.ca (S.H.J.H.); 6 Department of Supportive Care, Princess Margaret Cancer Centre, Toronto, ON M5G 2M9, Canada; jackie.bender@uhnresearch.ca (J.B.);; 7 Department of Psychology, BC Children’s Hospital, Vancouver, BC V6H 3N1, Canada; 8 Young Adult Cancer Canada, St. John’s, NL A1B 3K3, Canada; 9 Discipline of Oncology, Faculty of Medicine, Memorial University, St. John’s, NL A1C 5S7, Canada

**Keywords:** oncology, adolescents, young adults, AYA, coping

## Abstract

A greater understanding of how young people cope with a cancer diagnosis is needed in order to inform age-appropriate supportive care. This paper describes the coping strategies used and explores relationships between coping strategies and personal, medical, and psychological variables among young adults (YAs) diagnosed with cancer. YAs (*n* = 547, mean age = 34.05 ± 6.00 years) completed an online survey, including the Brief COPE and measures of psychological functioning. Descriptive statistics and bivariate correlations were computed. Acceptance, self-distraction, positive reframing, and planning were the most used coping strategies by this sample. There were small (*r* = −0.09) to large (*r* = 0.51) significant relationships between personal, medical, and psychological variables and selected coping strategies. Coping with a cancer diagnosis early in life remains poorly understood. Identifying additional correlates and exploring inter- and intrapersonal variation in coping strategy use is required.

## 1. Introduction

While relatively rare, cancer among adolescents and young adults (AYAs; 15 to 39 years of age) is associated with negative disease- and treatment-related effects that harm all health domains [1,2,3]. Uniquely for AYAs, cancer coincides with a pivotal developmental period [4], which can exacerbate or precipitate adverse effects, requiring age-appropriate supportive care to help AYAs cope with their disease [2].

Coping, conceptualized as efforts to manage demands perceived as exceeding one’s resources [5], has been relatively unexplored among AYAs diagnosed with cancer. The limited literature on coping among AYAs diagnosed with cancer is tenuous (e.g., small sample sizes, researcher-generated questionnaires) and fraught with heterogeneity (e.g., various questionnaires used, different coping approaches examined), making it difficult to draw firm conclusions [6,7,8]. Further, the specific coping strategies that are (and are not) being used by this cohort vary across quantitative and qualitative investigations [6,9,10,11]. Given that coping is complex and dynamic across the lifespan [5], identifying the strategies AYAs diagnosed with cancer use to cope with their disease could offer insights to inform age-appropriate supportive care. 

Additionally, there is a need to identify those AYAs who may be more/less likely to use selected strategies and experience positive or negative outcomes in the wake of their cancer diagnosis. While there is evidence among older adults affected by cancer to suggest select coping strategies and styles may be related to personal (e.g., age, sex), medical (e.g., treatment status, time since diagnosis), and psychological variables (e.g., emotional distress, post-traumatic growth [PTG]) [12,13], such relationships have yet to be examined among AYAs diagnosed with cancer. Thus, the objectives of this sub-study were to describe coping and estimate relationships between coping strategies and potentially relevant personal (age, sex), medical (treatment status, time since diagnosis), and psychological (post-traumatic growth, distress) variables in this cohort.

## 2. Materials and Methods

The data analyzed and reported herein were collected as part of a larger observational study using an online survey: the Young Adults with Cancer in Their Prime (YACPRIME) study [1,2,3]. 

### 2.1. Participants

Young adults (YAs) were eligible if they were ≥19 years, had received a cancer diagnosis as an AYA, and were Canadian residents. 

### 2.2. Procedures

Relevant ethical approval was obtained, and YAs were recruited through advertising, social media, healthcare provider referral, and the Young Adult Cancer Canada (YACC) network. Following informed consent, participants completed the online survey. Further details covering the participants and procedures have been published elsewhere [1,2,3].

### 2.3. Measures

#### 2.3.1. Personal and Medical Information

A researcher-generated questionnaire collected information covering age, sex, ethnicity, location (rural or urban), cancer type, treatment status (on- or off-treatment), and time since diagnosis. 

#### 2.3.2. Coping

The Brief COPE was used to measure coping [14], where 28 items assess 14 coping strategies. Each item is rated on a 4-point Likert-type scale ranging from 1 (*I haven’t been doing this at all*) to 4 (*I have been doing this a lot*). Coping strategy scores were computed by summing relevant items (see Supplementary File S1), with higher scores indicating greater use. Correlations between items on each subscale in this sample ranged from r = 0.32 (self-distraction) to r = 0.92 (substance use). 

#### 2.3.3. Psychological Variables

The 10-item Kessler Psychological Distress Scale (K10) was used to measure distress [15]. The K10 employs a Likert-type scale ranging from 1 (*none of the time*) to 5 (*all of the time*). A global distress score was calculated by summing scores, with higher scores indicating greater distress (α in this sample = 0.91). The PTG Inventory (PTG-I) was used to assess PTG [16]. The PTG-I comprises 21 items measuring 5 subdimensions of post-traumatic growth. Each item is rated on a 6-point Likert scale ranging from 0 (*I did not experience this change*) to 5 (*I experienced this change to a very great degree*). A total PTG score was calculated by summing all responses, with higher scores representing greater PTG (α in this sample = 0.92).

### 2.4. Data Analysis

Data were analyzed using SPSS (version 27). Data were first inspected for missing values, and after removing participants with missing data on the variables of interest (n = 75), the data were screened to ensure that the assumptions for planned analyses were met. Normally distributed data were described by mean and standard deviation (SD), and binary and categorical variables were presented using frequency and percentages. Bivariate correlations (Pearson and point-biserial; *p* < 0.05) were estimated to explore the relationships between the 14 coping strategies and personal, medical, and psychological variables. 

## 3. Results

The sample consisted of 547 YAs, who were, on average, 34.05 (SD = 6.00) years of age, and who had been diagnosed with cancer an average of 4.89 (SD = 5.29) years prior to survey completion. The majority of participants self-identified as female (n = 475; 86.8%), white (n = 477; 87.2%), and as residing in an urban location (n = 404; 73.9%). Most of the sample had been diagnosed with breast cancer (n = 148; 27.1%) or a hematological malignancy (n = 147; 26.8%). The remainder had been diagnosed with genitourinary and gynecologic (n = 65; 11.9%), thyroid (n = 44; 8.0%), brain (n = 31; 5.7%), head and neck (n = 9; 1.6%), gastrointestinal (n = 45; 8.2%), skin (n = 15; 2.7%), other types of cancer (e.g., bone tumor, neuroendocrine cancer; n = 38; 6.9%), or multiple types of cancer (n = 5; 0.9%). 

Participants’ scores on the variables of interest for this sub-study are shown in Table 1. The coping strategies used most often in this sample were self-distraction (e.g., turning to work or other activities to think about it less), active coping (e.g., taking action and concentrating one’s efforts towards doing something), emotional support (e.g., getting emotional support, comfort, and understanding from others), positive reframing (e.g., looking for something good in the situation), planning (e.g., coming up with a strategy or way forward), and acceptance (e.g., learning to live with it). Substance use (e.g., using alcohol or other drugs to make one feel better), behavioral disengagement (e.g., giving up trying to deal with it), and religion (e.g., praying or meditating) were the least often used strategies. Of note, participants’ scores on distress and PTG were moderate, relative to score ranges.

The bivariate correlations between coping strategies and personal, medical, and psychological variables are presented in Table 2. There were small, significant associations between age and sex and selected coping strategies. Specifically, as age increased, the use of self-distraction (r = −0.18), humor (r = −0.17), self-blame (r = −0.11), and instrumental support (r = −0.09) decreased. Females were more likely than males to use emotional support (r_pb_ = 0.15), religion (r_pb_ = 0.10), and instrumental support (r_pb_ = 0.10) and less likely to use behavioral disengagement (r_pb_ = −0.10) to help them cope. In terms of medical variables, there were small, significant associations between treatment status and time since treatment, such that being on-treatment was associated with greater use of denial (r_pb_ = 0.11) and venting (r_pb_ = 0.11). And, as time from diagnosis increased, the use of denial (r = −0.09), emotional support (r = −0.13), venting (r = −0.13), humor (r = −0.14), and instrumental support (r = −0.11) decreased. Finally, there were small, moderate, and large significant relationships between distress and PTG and selected coping strategies. Indeed, higher levels of distress were associated with greater use of denial (r = 0.37), behavioral disengagement (r = 0.51), self-blame (r = 0.51), self-distraction (r = 0.21), substance use (r = 0.25), venting (r = 0.22), and humor (r = 0.09), and less use of emotional support (r = −0.11) and acceptance (r = −0.14). Greater PTG was significantly related to greater use of active coping (r = 0.39), positive reframing (r = 0.47), planning (r = 0.34), acceptance (r = 0.36), religion (r = 0.49), instrumental support (r = 0.32), self-distraction (r = 0.09), emotional support (r = 0.27), and venting (r = 0.15). Conversely, less PTG was associated with greater substance use (r = −0.09) and behavioral disengagement (r = −0.21) as coping strategies. The remaining relationships were small (rs = 0.00–0.08) and not statistically significant (ps > 0.05).

## 4. Discussion

This study sought to describe coping strategies and estimate relationships between coping strategies and relevant personal, medical, and psychological variables. Aligned with prior research, diagnosis acceptance, support seeking, reframing, and distraction were endorsed [9,11,17]. However, participants in this sample also reported planning and taking action (i.e., active coping), which have been reported less in research with young adults (21–39 years) and older samples (40–79 years) [6]. Religion, substance use, and behavioral disengagement were among the least used strategies in this sample, which also stands in contrast to prior research with adults (32–60 years) [18] and AYAs diagnosed with cancer [19].

Age was negatively related to self-distraction, humor, and self-blame, such that older participants reported using these strategies less. This differs from prior work that suggests avoidant and distracting coping styles increase with age [20,21,22,23]. Given that coping is a dynamic construct that changes over time and with life experiences, exploring the developmental trajectories of varied coping strategies could offer insights into the needs and preferences of each life stage and provide a foundation to tailor care appropriately for patients at different ages.

In this study, females were more likely to use emotional support, religion, and instrumental support and less likely to use behavior disengagement. One possible explanation for this could relate to existing gender stereotypes, which may make it easier for the females in this sample to share information about their diagnosis and seek support from others [24]. There were also differences in coping strategies based on treatment status and time since treatment, which supports findings from Miedema and colleagues [10]. Notwithstanding the patterns observed herein, it is important to note that the strength of the relationships between personal and medical variables and selected coping strategies were variable. Considering the highly individualized nature of coping [10], further efforts are required to explore intra- and interpersonal differences in coping. Primary and allied healthcare providers should exert caution when extrapolating these findings to their work with patients. 

Greater use of denial, behavioral disengagement, self-blame, self-distraction, substance use, venting, and humor was related to greater distress. However, the mechanisms underlying this relationship, including whether the relationship is bidirectional, are unknown. Exploring coping strategies and psychological outcomes via prospective observational studies and structural equation modeling (e.g., cross-lagged panel analysis) could provide insight into how coping strategy use develops and changes, which could lay a foundation for intervention efforts to better support coping and mitigate distress in this cohort.

More frequent use of active coping, positive reframing, planning, acceptance, religion, emotional support, and instrumental support was related to PTG, which may be due in part to the conceptual overlap between PTG and coping. Tedeschi and Calhoun [25] suggest that PTG describes the positive changes that result from coping with a stressful life event such as cancer. Again, the longitudinal pathways of this relationship are not well understood among AYA cancer. Similar to above, further research is required to better understand coping and PTG. 

Interestingly, self-distraction and venting were both positively related to distress and PTG. This finding is notable given that many researchers have conflated these strategies and reported a composite score representing maladaptive coping [17]. Results from this study suggest that these strategies are related in the same way to positive and negative psychological outcomes, suggesting that the strategies in and of themselves may not be inherently adaptive or maladaptive. Rather, whether the use of self-distraction and venting are helpful or not may relate to other individual factors such as social support, over reliance on these coping techniques, or other factors. 

Though this study contributes valuable information about coping among AYAs diagnosed with cancer, the findings should be viewed within the context of their limitations. This larger study recruited a large, representative (in terms of cancer diagnosis) sample of young adults diagnosed with cancer as AYAs. However, it was homogenous regarding sociodemographic variables (e.g., education, sex, race/ethnicity, rurality), limiting the ability to perform sub-group analyses and better understand coping among those who have been historically under-represented in the literature. Additionally, participants were primarily recruited and completed the survey online, which could have excluded those with lower technology literacy or access. Targeted recruitment is needed to reach hard-to-access samples. Finally, though the Brief COPE (a popular questionnaire for assessing coping) was used, comparing findings from this study and published research reports was challenging as most researchers report composite scores (not scores on each subscale). The study team made the decision to report subscale scores only to better advance the study of coping by exploring unique strategies used by this population and to adhere to guidance from developers of the Brief COPE who have not tested or validated composite scores. 

Coping is an integral part of the cancer journey. Participants in this study report using a wide range of strategies at different points in their experience, which were related in different ways to personal, medical, and psychological variables. The findings highlight several avenues of future inquiry and call attention to the need for age-appropriate, individualized care. 

## Figures and Tables

**Table 1:** Participant demographics and scores on main study variables.

Variable	Score Range	Mean (SD)
Age	--	34.05 (6.00)
Sex		
Female **^†^**	--	475 (86.8)
Treatment status		
On-treatment **^†^**	--	167 (30.5)
Time since diagnosis	--	4.89 (5.29)
Distress	10–50	24.64 (7.91)
PTG	0–105	57.24 (21.97)
Coping		
Self-distraction	1–8	5.75 (1.60)
Active coping	1–8	5.32 (1.58)
Denial	1–8	2.81 (1.33)
Substance use	1–8	3.09 (1.70)
Emotional support	1–8	5.27 (1.70)
Behavioral disengagement	1–8	2.93 (1.31)
Venting	1–8	4.40 (1.56)
Positive reframing	1–8	5.36 (1.78)
Planning	1–8	5.41 (1.68)
Humor	1–8	4.78 (1.96)
Acceptance	1–8	6.13 (1.53)
Religion	1–8	3.81 (1.93)
Self-blame	1–8	4.12 (1.74)
Instrumental support	1–8	4.64 (1.67)

Notes. PTG = post-traumatic growth; SD = standard deviation. **^†^** indicates that data is presented as n (%).

**Table 2:** Correlations between coping and personal, medical, and psychological variables.

Variables	1	2	3	4	5	6	7	8	9	10	11	12	13	14	15	16	17	18	19
1. Age	--																		
2. Sex ^†,‡^	−0.03	--																	
3. Treatment status ^†,^ˆ	−0.06	0.094 *	--																
4. Time since treatment	**0.47**	**−0.14**	**−0.28**	--															
5. Distress	−0.10 *	0.03	0.11 *	**−0.18**	--														
6. PTG	0.04	0.07	−0.07	**0.16**	**−0.13**	--													
7. Self-distraction	**−0.18**	0.08	−0.01	−0.08	**0.21**	0.09 *	--												
8. Active coping	0.02	0.08	0.02	−0.01	−0.07	**0.39**	**0.28**	--											
9. Denial	0.00	0.01	0.11 *	−0.09 *	**0.37**	0.00	**0.12**	−0.00	--										
10. Substance use	−0.01	−0.07	−0.08	0.00	**0.25**	−0.09 *	**0.11**	−0.05	**0.22**	--									
11. Emotional support	−0.08	**0.15**	0.07	**−0.13**	−0.11 *	**0.27**	**0.17**	**0.33**	−0.10 *	−0.09 *	--								
12. Behavioral disengage	−0.05	−0.10 *	−0.05	−0.02	**0.51**	**−0.21**	0.08	**−0.22**	**0.37**	**0.26**	**−0.21**	--							
13. Venting	−0.08	0.04	0.11 *	**−0.13**	**0.22**	**0.15**	**0.18**	**0.16**	**0.14**	**0.14**	**0.41**	**0.13**	--						
14. Positive reframing	−0.03	**0.15**	−0.04	−0.03	−0.07	**0.47**	**0.18**	**0.49**	0.05	−0.01	**0.36**	**−0.23**	**0.21**	--					
15. Planning	0.01	0.07	0.04	−0.04	0.07	**0.34**	**0.26**	**0.59**	0.05	−0.03	**0.31**	−0.10 *	**0.27**	**0.51**	--				
16. Humour	**−0.17**	0.02	0.05	**−0.14**	0.09 *	0.05	**0.19**	**0.15**	−0.02	0.06	**0.17**	−0.02	**0.18**	**0.28**	**0.21**	--			
17. Acceptance	0.02	0.03	0.02	0.00	**−0.14**	**0.36**	**0.16**	**0.40**	**−0.23**	−0.06	**0.27**	**−0.22**	**0.17**	**0.44**	**0.44**	**0.25**	--		
18. Religion	0.06	0.10 *	−0.07	0.01	0.012	**0.49**	0.06	**0.29**	0.04	−0.06	**0.21**	−0.08	**0.14**	**0.37**	**0.26**	−0.03	**0.25**	--	
19. Self-blame	**−0.11**	0.01	−0.04	−0.08	**0.51**	−0.03	**0.21**	−0.04	**0.30**	**0.26**	−0.03	**0.46**	**0.26**	−0.04	**0.13**	0.05	−0.10 *	0.06	--
20. Instrumental support	−0.09 *	0.10 *	0.03	**−0.11**	−0.04	**0.32**	**0.18**	**0.39**	−0.02	**−0.12**	**0.69**	**−0.14**	**0.41**	**0.36**	**0.40**	**0.18**	**0.26**	**0.28**	−0.01

Notes. behav disengage = behavioral disengagement, PTG = post-traumatic growth. ^†^ = point-biserial correlation, ^‡^ 1 = male, 2 = female, ˆ 1 = on-treatment, 2 = off-treatment. * *p* < 0.05; *p* < 0.01 are indicated in bold.

## Data Availability

Data will be available to investigators whose proposed use of the data has been approved by an independent review committee. To gain access, data requestors will need to sign a data access agreement.

## Supplementary Materials

The following supporting information can be downloaded at: https://www.mdpi.com/article/10.3390/curroncol31020050/s1. Supplementary File S1: Items and information on the scoring of the Brief COPE.
