# Supplementary material for: Describing and Exploring Coping Strategies among Those Diagnosed with Cancer as an Adolescent or Young Adult: A YACPRIME Study
Source: Curr Oncol. 2024 Jan 24;31(2):685–92. doi: 10.3390/curroncol31020050 (PMC10888435; doi:10.3390/curroncol31020050)
Supplement: Supplementary file 1 [file curroncol-31-00050-s001.zip › curroncol-2759291-supplementary.pdf]

Supplementary File 1. Brief COPE Scoring

Brief COPE items

1. I've been turning to work or other activities to take my mind off things.
2. I've been concentrating my efforts on doing something about the situation I'm in.
3. I've been saying to myself "this isn't real.".
4. I've been using alcohol or other drugs to make myself feel better.
5. I've been getting emotional support from others.
6. I've been giving up trying to deal with it.
7. I've been taking action to try to make the situation better.
8. I've been refusing to believe that it has happened.
9. I've been saying things to let my unpleasant feelings escape.
10. I’ve been getting help and advice from other people.
11. I've been using alcohol or other drugs to help me get through it.
12. I've been trying to see it in a different light, to make it seem more positive.
13. I’ve been criticizing myself.
14. I've been trying to come up with a strategy about what to do.
15. I've been getting comfort and understanding from someone.
16. I've been giving up the attempt to cope.
17. I've been looking for something good in what is happening.
18. I've been making jokes about it.
19. I've been doing something to think about it less, such as going to movies, watching TV, reading, daydreaming, sleeping, or shopping.
20. I've been accepting the reality of the fact that it has happened.
21. I've been expressing my negative feelings.
22. I've been trying to find comfort in my religion or spiritual beliefs.
23. I’ve been trying to get advice or help from other people about what to do.
24. I've been learning to live with it.
25. I've been thinking hard about what steps to take.
26. I’ve been blaming myself for things that happened.
27. I've been praying or meditating.
28. I've been making fun of the situation.

Original scoring approach (14 subscales)

Self-distraction: items 1, 19
Active coping: items 2, 7
Denial: items 3, 8
Substance use: items 4, 11
Use of emotional support: items 5, 15
Behavioral disengagement: items 6, 16
Venting: items 9, 21
Positive reframing: items 12, 17
Planning: items 14, 25
Humor: items 18, 28

Acceptance: items 20, 24
Religion: items 22, 27
Self-blame: items 13, 26
Use of instrumental support: items 10, 23
